# Analysis of the Impact of External Debt on Health in an Emerging Asian Economy: Does FDI Matter?

**DOI:** 10.3389/fpubh.2022.824073

**Published:** 2022-01-31

**Authors:** Yechi Ma, Mengyun Hu, Quratulain Zafar

**Affiliations:** ^1^School of Business, Northeast Normal University, Jilin, China; ^2^School of Business, Changchun Guanghua University, Jilin, China; ^3^UCP Business School, University of Central Punjab, Lahore, Pakistan

**Keywords:** debt, FDI, health, expenditure, economy

## Abstract

In this study, our main objective is to find the impact of FDI and external debt on health outcomes in emerging Asian economies from 1991 to 2019. To that end, we have collected data for seven economies: Bangladesh, Malaysia, Philippines, Thailand, Sri Lanka, China, and India. We have relied on the panel ARDL (PARDL) method for empirical analysis. The study's findings confirmed that the debt has increased infant mortality and decreased life expectancy in emerging Asian economies in the long run. On the other side, the FDI causes infant mortality to fall and life expectancy to rise in the long run in emerging Asian economies. Similarly, the health expenditures also reduced the infant mortality rate, though the impact is insignificant, and improved the life expectancy in emerging Asian economies. The causal analysis confirmed the two-way causality between health expenditure, infant mortality, and health expenditure and debt.

## Introduction

Since the last few decades, a sharp increase has been observed in external debt in several economies (1). The external debt grew sharply in Europe, the US, emerging economies, and most industrialized economies (2). Existing literature reports that as well as socio-economic determinants (for instance, wealth, income, and education), debt also contribute significantly in effecting health consequences (3). Alola and Ozturk (4) noted that health is wealth and financial stress severely affects the health of the population. A high degree of debt repayments can behave as an active source of anxiety that leads to poor physical and mental health and psychological distress that in turn might worsen overall welfare (5, 6). Such stresses instigated by the debt effect can tend to enhance unhealthy behaviors, like poor dietary habits, alcohol consumption, and smoking (7). Indebted economies tend to enlarge additional debt to pay for good quality health and food care services to the masses. Debt accumulation can minimize the accessibility of forthcoming resources for health-related investments and tend to a vicious phase, where largely accumulated debt could be a consequence and cause of poor health conditions (3).

The previous stock of literature provides limited evidence, demonstrating that short-term debt significantly influences health outcomes compared to long-term debt (8). The comparative measure of debt burden is often considered a signal of financial sector vulnerability (9). Previous studies used to measure health outcomes using two common proxies: premature mortality and life expectancy rates at birth (10, 11). Numerous studies have explored the association between government expenditures and revenues and health outcomes in developing economies, and several related health outcomes with the factors of government expenditures (12, 13). Health financing is a core factor that can be undertaken to improve the economy's economic growth and human development (14). Most studies have considered the nexus between external aid inflows and fiscal implications (15, 16).

Literature highlights the historical significance of aid and external debt flows, specifically in aid relying on Asian economies. However, it is worth mentioning that a substantial portion of foreign aid consists of loans and, thus, increased external debt. Therefore, isolation of external debt influence is significant in its own right. The current stock of literature, thus, usually does not consider this perspective. Very little stock of literature, such as the study done by Cashel-Cordo and Craig (17), focuses on the effect of aid but comprises debt servicing among the variables measuring revenues and government expenditures. However, the study reports the negative influence of debt service and has not isolated the health component's effect. Likewise, Mahdavi (18) study explores the impact of the external debt on the structure of government spending but does not separate health expenditure. Fosu (19) study examines the effect of external debt on education. Similarly, various studies have explored the determinants of health outcomes but did not relate it with the significance of external debt (20).

Generally, investment is considered a major driver of economic development and growth (21). However, only domestic investment is not enough to attain sustainable development and growth. For instance, in 2018, United Nations Conference on Trade and Development noted that there were few governing constraints on FDI, as according to new investment policies by economies support trade liberalization, promotion, and facilitation, through abolishing and reducing constraints on entry, streamlining administrative processes for foreign investors and providing fiscal inducements for investments that are industry or region-specific (22). However, the effect of FDI on development and growth still remains debatable. Growth is subject to both social and physical capital (23). On one side, inflows of FDI can enlarge the transfer of technology and domestic capital, resulting in higher incomes and economic growth. Due to FDI inflow, income level increases, raising private and public expenditures on health inputs (for example, medical care, nutritious food, improved sanitation, and water), resulting in improved health outcomes. Furthermore, FDI inflow in the health sector can enhance the provision of medical-related goods and services, reducing prices, thus leading to a further increase in demand for such goods and services (24–26).

A vast literature reported that FDI results in decreasing income inequality which suggests that now poor households can afford health care and necessities (27, 28). Additionally, FDI inflows that directly go to the manufacturing sector enhance negative externalities that reduce environmental quality in the recipient economies and negatively influence health outcomes. Hitam and Borhan (29) study reported that FDI inflow deteriorates environmental quality. Agosin and Machado (30) revealed that FDI results in crowing out of the domestic investment. Crowing out of domestic investment tends to increase unemployment, reducing the affordability of basic necessities and healthcare. Gilmore and McKee (31) reported that FDI increases the consumption and production of harmful products like tobacco that can be damaging to health. Furthermore, the study of Herzer and Nunnenkamp (32) found that FDI put competitive and uncertain pressure on workers, leading to stress, which ultimately affects health outcomes. A bulk of literature (33, 34) has mainly focused on the impact of FDI on economic development, with very limited studies having researched on impact of FDI on health outcomes (26, 35).

In light of the above background, it is obvious that vast literature is available on external debt and health nexus, and very literature is found on FDI and health nexus. However, none of the studies has explored the simultaneous impact of external debt and FDI on health outcomes. To fill the existing vacuum present study aims to explore the effects of external debt and FDI on health outcomes in an emerging Asian economy. The study investigates the association between external debt, FDI and health outcomes by adopting mortality rate and life expectancy proxy measures for health outcomes. The study's outcomes should facilitate government agencies with the execution and design of policy initiatives that target outcomes of health in coincidence with debt advice, FDI utilization, and financial literacy programs to assist management in managing FDI and external debt-related issues better.

## Model and Methods

It is observed that an increase in public debt has negative effects on the economy (36). This, in turn, could lead to a decline in health outcomes (37). Similarly, FDI is considered another important source of health. FDI inflows can enlarge domestic capital and transfer technology, resulting in health outcomes. Moreover, FDI may affect economic growth and other important indicators, such as health outcomes. FDI could positively impact health mainly by increasing the demand for health-related goods and services in host economies. According to the literature, debt and FDI are the most important emerging determinants of health outcomes. Following recent literature, e.g., (Clayton et al. and Immurana) (26, 37), we adopt the following long-run health econometric model for emerging Asian economies:


(1) $$\begin{array}{l} Health_{it}=\;\varphi_{0}+\;\varphi_{1}Debt_{it}\\ \;+\;\varphi_{2}FDI_{it}+\;\varphi_{3}HE_{it}+\varphi_{4}GDP_{it}+\varphi_{5}Trade_{it}+\varepsilon_{it} \end{array}$$

Where health outcomes (Health) is dependent on the public debt (debt), foreign direct investment (FDI), health expenditure (HE), GDP per capita (GDP), trade openness (Trade), and randomly distributed error term (ε_*it*_). Debt worsens health outcomes, but FDI is the driving force of health. Thus, we expect an estimate of φ_1_ to be negative and φ_2_ to be positive. However, this equation only provides a long-run estimate, and to get short-run estimates, we will reproduce equation (1) in error correction format as shown below:


(2) $$\begin{array}{l} {\Delta Health}_{it}=\alpha_{0}+\;\overset{p}{\underset{i=1}{\sum }}\pi_{i}Debt_{it-i}+\;\overset{p}{\underset{i=0}{\sum }}\psi_{i}{\Delta FDI}_{it-i}\\ \;+\overset{p}{\underset{i=0}{\sum }}u_{i}{\Delta HE}_{it-i}+\;\overset{p}{\underset{i=0}{\sum }}\theta_{i}GDP_{it-i}+\;\overset{p}{\underset{i=0}{\sum }}\lambda_{i}Trade_{it-i}\\ \;+\;\omega_{1}Health_{it-1}+\;\omega_{2}Debt_{it-1}+\;\omega_{3}FDI_{2,it-1}\\ \;+\;\omega_{4}HE_{it-1}+\omega_{5}GDP_{it-1}+\omega_{5}Trade_{it-1}\\ \;+\lambda .ECM_{it-1}+\;\varepsilon_{it} \end{array}$$

Specification (2) is known as the panel ARDL of (38, 39). This method can provide short and long-run estimates simultaneously. The short and long-run estimates can be measured using ARDL-PMG and ARDL-MG and choosing the most efficient method via the Hausman test.

The estimates connected to difference indicator (Δ) are short-run estimates, and the estimates attached to ω_2_ − ω_5_ normalized on ω_1_represent the long-run estimates. The long-run estimates are valid if they are co-integrated. This approach contains two steps for assessing the long-run nexus. The first step is to scrutinize the existence of co-integration among variables, but the second step is to estimate the long-run coefficients. To that end, we need to check if the estimate of ECM_t−1_ is negatively significant or not. Another advantage of this method is that it can work well even if the sample size is small. Further, this method can also consider the integrating properties of the variables and estimate whether they are I(0), I(1), or a blend of both. We use panel Granger causality tests to perform causality nexus among concern variables.

## Data

The study determines to examine the impact of external debt and FDI on health conditions of selected emerging Asian economies for the period 1991–2019. These economies include Bangladesh, China, India, Malaysia, the Philippines, Sri Lanka, and Thailand. Table 1 provides detailed information regarding symbols, definitions, and data sources. The dependent variable, health condition, is measured by infant mortality (per 1,000 live births) and life expectancy (at birth, in total years). Focused variables of the study are central government debt (in total percent of GDP) and foreign direct investment (measured as net flows in percent of GDP). The study used GDP per capita (constant 2015 US$), trade openness (total trade in percent of GDP), and health expenditures (in percent of GDP) as control variables. Required data on all variables have been collected from the World Bank and IMF.

**Table 1:** Definitions and sources.

**Variables**	**Symbol**	**Definitions**	**Sources**
Infant mortality	IM	Mortality rate, infant (per 1,000 live births)	World bank
Life expectancy	LE	Life expectancy at birth, total (years)	World bank
Government debt	Debt	Central government debt, total (% of GDP)	IMF
Foreign direct investment	FDI	Foreign direct investment, net inflows (% of GDP)	World bank
GDP per capita	GDP	GDP per capita (constant 2015 US$)	World bank
Trade openness	Trade	Trade (% of GDP)	World bank
Health expenditure	HE	Current health expenditure (% of GDP)	World bank

## Results and Discussion

This section reports the findings of cross-sectional dependence (CD) tests, unit root tests, co-integration tests, and panel ARDL regression for outcomes. As can be seen from Table 2, the null hypothesis could be rejected. Hence, it could be concluded that there exists CD in our analysis. Before the empirical investigation of external debt-FDI-health nexus, it is required to check the stationary properties of data. For that purpose, the study employed IPS and ADF tests. Table 2 reported the outcomes of these unit root tests. Findings of both unit root tests revealed that infant mortality, life expectancy, and FDI are stationary at level, i.e., I(0), and external debt, health expenditures, GDP, and trade are stationary at the first difference, i.e., I(1). These findings of unit root tests encouraged us to employ the ARDL-PMG technique to investigate further the impact of external debt and FDI on the health condition of selected emerging Asian economies. Table 3 provides the findings of health estimates of the ARDL-PMG approach.

**Table 2:** Cross-sectional dependence and unit root testing.

**Cross-sectional dependence test**
IM = f(Debt, FDI, GDP, Trade, HE)	1.881^*^		
LE = f(Debt, FDI, GDP, Trade, HE)	3.566^***^		
**Unit root testing**						
	**IPS**			**ADF**
	**I(0)**	**I(1)**	**Decision**	**I(0)**	**I(1)**	**Decision**
IM	−3.785^***^		I(0)	−3.025		I(0)
LE	−2.912^***^		I(0)	−1.965^**^		I(0)
Debt	−0.612	−4.955^***^	I(1)	−0.621	−4.956^***^	I(1)
FDI	−4.065^***^		I(0)	−3.987		I(0)
HE	1.965	−8.525^***^	I(1)	1.564	−7.452^***^	I(1)
GDP	1.023	−3.623^***^	I(1)	1.010	−3.568^***^	I(1)
Trade	−0.071	−5.452^***^	I(1)	−0.070	−5.022^***^	I(1)

*** *p < 0.01;*

** *p < 0.05; and*

* *p < 0.1*.

**Table 3:** Long and short-run estimates of health.

	**Infant mortality**				**Life expectancy**			
	**Coefficient**	**Std. error**	**t-Statistic**	Prob.^*^	**Coefficient**	**Std. error**	**t-Statistic**	Prob.^*^
**Long-run**								
DEBT	0.040^***^	0.008	5.276	0.000	−0.033^***^	0.002	13.43	0.000
FDI	−0.277^***^	0.069	4.035	0.000	0.210^***^	0.033	6.390	0.000
HE	−0.113	0.195	0.578	0.564	0.476^***^	0.124	3.854	0.000
GDP	−11.15^***^	0.452	24.64	0.000	7.818^***^	0.143	54.71	0.000
TRADE	−0.715	0.539	1.327	0.187	1.869^***^	0.236	7.936	0.000
**Short-run**								
D(DEBT)	0.010^**^	0.005	2.000	0.050	0.001	0.001	0.505	0.615
D(DEBT(−1))					0.001	0.000	0.313	0.755
D(FDI)	0.009	0.016	0.530	0.597	0.007^*^	0.004	1.687	0.095
D(FDI(−1))					0.295	0.207	1.425	0.157
D(HE)	0.190	0.249	0.765	0.446	0.004	0.005	0.800	0.426
D(HE(−1))					0.016	0.010	1.602	0.112
D(GDP)	−1.800^*^	1.036	1.800	0.078	0.005^**^	0.002	2.572	0.012
D(GDP(−1))					0.295	0.207	1.425	0.157
D(TRADE)	0.401	0.488	0.821	0.413	0.076	0.072	1.057	0.293
D(TRADE(−1))					−0.121	0.088	1.382	0.170
C	19.65	13.96	1.408	0.161	0.108	0.077	1.407	0.162
**Diagnostics**								
Log-likelihood	202.4				638.1			
Kao-cointegration	−2.875^***^				−2.988^***^			
ECM(−1)	−0.189^*^	0.110	−1.800	0.078	−0.034^**^	0.017	−1.980	0.050

*** *p < 0.01;*

** *p < 0.05; and*

* *p < 0.1*.

In the long-run, the debt variable exhibits a significant and positive influence on infant mortality, revealing that the infant mortality rate increases due to external debt. The coefficient estimate displays that due to a one percent upsurge in external debt, the infant mortality rate increases by 0.040 percent in the long run. However, FDI exerts a significant and negative impact on the infant mortality rate, depicting that the infant mortality rate declines significantly due to the increased inflow of FDI. The coefficient estimate shows that in response to a one percent increase in FDI inflow, the infant mortality rate decreased by 0.277 percent in the long run. GDP also exerts a significant and negative impact on the infant mortality rate, implying that due to a one percent increase in GDP, the infant mortality rate reduces by 11.15 percent in the long-run. Short-run findings of this model display that external debt results in increasing infant mortality rate; however, FDI produces a statistically insignificant impact on infant mortality rate. In terms of control variables, only GDP results in a declining infant mortality rate in the short-run, the other two variables (trade and health expenditures) exhibit insignificant impact on infant mortality rate.

The long-run findings of the life expectancy model demonstrate that external debt has a significant and negative impact on life expectancy, revealing that due to an increase in external debt, life expectancy tends to decline significantly. It is apparent from the coefficient estimate that due to a one percent increase in external debt, life expectancy tends to decline by 0.033 percent in the long run. The empirical findings suggest a negative impact of external debt on health outcomes across economies. Findings suggest that external debt results in an increasing infant mortality rate and tends to decline life expectancy. The findings can be justified as the government is not quickly responsive to health care services in high debt. Moreover, external debt exerts negative pressure on the income level that reduces the capability of people to afford good quality health services. External debt is considered as stressful and directly or indirectly affects everyday life and health-related goods and services. This finding is also reliable with Shabbir and Yasin (40), who reported that debt servicing unfavorably influences education and health sector allocations and under process development projects. Thus, debt servicing may harmfully influence fiscal budget compositions in low-income economies. While external debt also affects poverty but it has a negative impact on health in emerging Asian economies. A similar finding is also reported by Loko et al. (41) for low-income economies.

In contrast, FDI reported a significant and positive impact on life expectancy, confirming that life expectancy tends to rise in a sample of selected emerging Asian economies due to an upsurge in FDI inflow. It is found that due to a one percent increase in the inflow of FDI, life expectancy tends to increase by 0.210 percent in the long run. The positive sign of FDI on health is that FDI inflows create economic growth and increase the level of income, which enables people to afford more health care services, henceforth indicating the enhanced quality of health (25, 30, 31, 42). Moreover, foreign organizations investing in a country may enhance health-related exercises like free medical care and health screening as part of corporate social responsibilities that improve health outcomes. FDI also tends to increase environmental quality, resulting in rich health outcomes. FDI increases income levels by generating employment opportunities that increase expenditures on health-enhancing goods and services. Additionally, the direct inflow of FDI in the health sector may enhance the provision of health-related goods and services at lesser costs that enhance the demand for health-related inputs, hence improving the health outcomes. These findings are in line with (30, 43, 44). FDI significantly increases the resilience of health systems in emerging Asian economies. The FDI enhances the capability of the health system, reduces the burden on public finances, and enhances the choice and quality for households of the host economy who can obtain private health care services that deteriorate income inequality (24).

As far as control variables are concerned, it is reported that in the long-run, health expenditures, GDP, and trade result in significantly increased life expectancy by 0.476 percent, 7.818 percent, and 1.869 percent, respectively. The short-run findings of this model show that external debt exerts no significant impact on life expectancy, but FDI results in enhancing life expectancy. In the case of control variables, only GDP has a significant and positive effect on life expectancy; coefficient estimates of health expenditures and trade variables are statistically insignificant in the short-run.

In the lower panel of Table 3, the results of some important diagnostic tests are given. These tests are imperative to confirm the findings of the ARDL model. These diagnostic tests include the Log-likelihood ratio, Kao-cointegration, and ECM. The findings of Log-likelihood tests confirm the goodness of fit of both models. There exists long-run co-integration among variables in both models, as depicted by the findings of the Kao-cointegration test. The statistically significant and negative coefficient estimates of the ECM test in both models confirm that there exists long-run co-integration and a tendency of convergence among variables toward achieving equilibrium. In Table 4, the causal analysis confirmed the two-way causality between health expenditure and infant mortality, and then health expenditure and debt. The results show no causality between FDI and IM, DEBT and LE, FDI and LE. In contrast, unidirectional causality exists from DEBT to IM. Also, no bidirectional causal nexus exists between focused and outcome variables.

**Table 4:** Panel causality results.

**Null hypothesis**	**F-Statistic**	**Prob**.	**Null hypothesis**	**F-Statistic**	**Prob**.
DEBT → IM	2.421^*^	0.091	DEBT → LE	2.323	0.100
IM → DEBT	1.257	0.287	LE → DEBT	0.068	0.934
FDI → IM	0.769	0.465	FDI → LE	1.139	0.322
IM → FDI	0.914	0.403	LE → FDI	1.468	0.233
HE → IM	5.622^***^	0.004	HE → LE	0.938	0.393
IM → HE	3.048^**^	0.050	LE → HE	2.893^*^	0.058
GDP → IM	1.266	0.284	GDP → LE	1.683	0.189
IM → GDP	1.389	0.252	LE → GDP	2.472^*^	0.087
TRADE → IM	2.548^*^	0.081	TRADE → LE	0.251	0.778
IM → TRADE	2.207	0.113	LE → TRADE	3.295^**^	0.039
FDI → DEBT	0.464	0.630	FDI → DEBT	0.464	0.630
DEBT → FDI	7.229^***^	0.001	DEBT → FDI	7.229^**^	0.001
HE → DEBT	2.422^*^	0.092	HE → DEBT	2.422^*^	0.092
DEBT → HE	3.665^**^	0.028	DEBT → HE	3.665^**^	0.028
GDP → DEBT	0.113	0.894	GDP → DEBT	0.113	0.894
DEBT → GDP	0.452	0.637	DEBT → GDP	0.452	0.637
TRADE → DEBT	0.832	0.437	TRADE → DEBT	0.832	0.437
DEBT → TRADE	0.187	0.830	DEBT → TRADE	0.187	0.830
HE → FDI	1.924	0.149	HE → FDI	1.924	0.149
FDI → HE	2.308	0.102	FDI → HE	2.308	0.102
GDP → FDI	1.645	0.196	GDP → FDI	1.645	0.196
FDI → GDP	1.806	0.167	FDI → GDP	1.806	0.167
TRADE → FDI	2.452	0.089	TRADE → FDI	2.452^*^	0.089
FDI → TRADE	0.528	0.591	FDI → TRADE	0.528	0.591
GDP → HE	4.368^*^	0.014	GDP → HE	4.368^**^	0.014
HE → GDP	4.469^*^	0.013	HE → GDP	4.469^**^	0.013
TRADE → HE	2.312	0.102	TRADE → HE	2.312	0.102
HE → TRADE	2.321	0.101	HE → TRADE	2.321	0.101
TRADE → GDP	3.994^**^	0.020	TRADE → GDP	3.994^**^	0.020
GDP → TRADE	0.815	0.444	GDP → TRADE	0.815	0.444

*** *p < 0.01;*

** *p < 0.05; and*

* *p < 0.1*.

## Conclusion and Implications

It is a well-known fact that economic growth is crucial for raising the living standard of the people by providing them with the best health and education facilities, employment opportunities, and social security benefits. Various factors can positively affect economic growth, and among them, FDI and external debt is crucial. Both these factors also have an important role in improving the health status of the people because the health status of the people is directly linked to the higher growth rate of the economies. Therefore, our main objective in this study is to find the impact of FDI and external debt on health outcomes in emerging Asian economies. To that end, we have collected data for seven economies: Bangladesh, Malaysia, Philippines, Thailand, Sri Lanka, China, and India. Our data was long enough, so we performed panel unit root tests and confirmed that we have a mixture of I(0) and I(1) variables; hence, we can apply panel ARDL-PMG.

The study's findings confirmed that the debt has increased infant mortality and decreased life expectancy in emerging Asian economies in the long run. On the other side, the FDI causes infant mortality to fall and life expectancy to rise in the long run in emerging Asian economies. Similarly, the health expenditures also reduce the infant mortality rate, though the impact is insignificant, and improve the life expectancy in emerging Asian economies. Lastly, the long-run relation between GDP and infant mortality is negative; whereas, positive between the GDP and life expectancy. In the short run, we observed mixed and conflicting findings. The causal analysis confirmed the two-way causality between health expenditure, infant mortality, and health expenditure and debt.

The findings are important in the context of emerging Asian economies, and we have provided some useful policy suggestions for the concerned stakeholders by relying on these findings. The first and foremost suggestion is that an appropriate level of debt can improve the economy's economic growth, which helps achieve a higher health status such as reduced infant mortality and life expectancy. However, higher debt may also put an extra burden on the economy in the form of higher debt servicing costs that may negatively impact the economy's GDP and, ultimately, health and other outcomes. Therefore, the Asian emerging economies should keep an eye on the country's total debt and don't let it increase too much because it could take away the financial resources from health and other sectors to the payment of original debt and related interest payments. Secondly, the Asian emerging economies should try to attract as much foreign investment as possible because it is considered an important factor in promoting economic growth and raising the living standard of the people. The FDI generates income that can be used to develop hospitals and other health facilities that reduces infant mortality rate and increase life expectancy.

## Data Availability Statement

Publicly available datasets were analyzed in this study. This data can be found at: https://data.worldbank.org/.

## Author Contributions

YM: conceptualization, software, data curation, and writing—original draft preparation. MH: methodology and writing—reviewing and editing. QZ: visualization and investigation. All authors contributed to the article and approved the submitted version.

## Funding

This study was supported by Research on the promotion of the international status of Chinese standards (Grant No. 19QT008).

## Conflict of Interest

The authors declare that the research was conducted in the absence of any commercial or financial relationships that could be construed as a potential conflict of interest.

## Publisher's Note

All claims expressed in this article are solely those of the authors and do not necessarily represent those of their affiliated organizations, or those of the publisher, the editors and the reviewers. Any product that may be evaluated in this article, or claim that may be made by its manufacturer, is not guaranteed or endorsed by the publisher.
